# Involvement of CC-chemokine receptor 2 in sepsis: focus on cognitive impairment

**DOI:** 10.1186/cc13005

**Published:** 2013-11-05

**Authors:** Mariana Gisely Amarante Teixeira da Cunha, Rachel Novaes Gomes, Daniele Bastos de Albuquerque, Daiane Chaves, Silvio Caetano Alves Júnior, Patrícia Reis, Rosália Mendez Otero, Patrícia Torres Bozza, Fernando Augusto Bozza, Hugo Caire Castro-Faria-Neto

**Affiliations:** 1 Laboratório Imunofarmacologia, Fiocruz-IOC, Rio de Janeiro, Brazil; 2 Fiocruz-IPEC, Rio de Janeiro, Brazil; 3 Lab. Neurobiologia Cellular e Molecular - CCS, Rio de Janeiro, Brazil

## Background

Sepsis is a major disease entity with important clinical implications. Critical illness survivors present long-term cognitive impairment, including problems with memory and learning. Chemokines are important to the recruitment of leukocytes to infectious tissue, but a few studies described the role of the CC-chemokine receptor 2 (CCL2) in the cognitive process. In this study, we analyze the involvement of CCR2 in the physiopathology of sepsis, especially in development of cognitive dysfunction.

## Materials and methods

The CCR2-deficient mice (CCR2^-/-^) were submitted to a CLP model and we analyzed the survival rate, the severity score of the animals during 144 hours and 15 days after the CLP, and we analyzed the memory of the animals. To analyze the contextual memory, the mice were submitted to the open field method and the water maze procedure. To evaluate the aversive memory, the passive avoidance test was performed.

## Results

First, we observed that the CLP group had cognitive impairment, but the CCR2^-/- ^group submitted to CLP had more severe cognitive impairment in comparison with the WT-CLP group. Interesting, the CCR2^-/- ^Sham group presented cognitive impairment, suggesting that CCR2 is important to the physiological process of cognition. We then submitted CCR2^-/- ^naive mice to water maze and passive avoidance tests. We found that CCR2^-/- ^naive mice have an impairment of aversive and contextual memory. The cognitive impairment was associated with a decrease of BDNF expression in the hippocampus. When we analyze the expression of β-amyloid protein in the brain of CCR2^-/- ^naive mice, we observed the increased in β-amyloid protein expression in the cortex and hippocampus of these animals, accompanied by increased cell proliferation in the dentate girus, and increased caspase-3 and caspase-12 expression in the hippocampus and cortex. We did not observe a difference in the numbers of neurons in the brain from CCR2^-/- ^naïve mice, as well the numbers of microglial cells. But, surprisingly, there was an increase of astrocytes in the hippocampus of CCR2^-/- ^mice.

## Conclusions

CCR2 is involved with the physiology of cognition, with the important role arising in the amyloid accumulation in the brain and induction of the caspase-3 pathway.

